# Ultrastructure, Polarity, and Reproduction of the Golgi Apparatus

**DOI:** 10.1111/boc.70053

**Published:** 2026-03-07

**Authors:** Bruno Goud

## Abstract

In this note published in 1957 in the *Proceedings of the French Academy of Sciences*, the zoologist Pierre‐Paul Grassé proposed for the first time that the cisternae of the Golgi apparatus form on the “cis” (proximal) face and are destroyed on the “trans” (distal) face to form chromophobe vesicles (presumably secretory granules), suggesting a model of maturation of the Golgi cisternae. This model is currently the most accepted by the cell biology community.

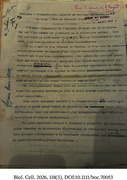

The fundamental element of the Golgi apparatus is the dictyosome, as described by the Classics of light microscopy. In earlier publications (Grassé et al. 1955; Grassé and Carasso 1957; Grassé 1956), either in collaboration or alone, I have described its true ultrastructure.

It is neither a bundle of tubules nor a cluster of lamellae. Each dictyosome consists of a stack of closed, flattened saccules whose outline is approximately quadrangular; the walls of the saccules are strongly osmiophilic, whereas their lumen appears electron‐lucent in all micrographs.

Any cluster of saccules constituting a dictyosome is accompanied by a procession of small vesicles, generally ellipsoidal, which are sufficiently osmiophilic to justify the term “osmiophilic vesicles”. Very numerous images have enabled us to establish that, from their edges, the saccules produce osmiophilic vesicles in variable quantities.

We have also observed the fragmentation of the saccules into a greater or lesser number of vesicles, differing only slightly—though generally larger—from the peripherally derived osmiophilic vesicles.

One important point remained to be clarified: up to now, photographs obtained by electron microscopy revealed no structure that could correspond to the chromophobe substance which adheres, in variable amount, to every dictyosome.

We realized that our failure to detect the chromophobe substance was due to the following two causes:

1. inadequate fixation, which does not preserve the said substance or renders it unrecognizable;
2. the very high proportion of sections that pass outside the chromophobe substance.

The examination of numerous ultrathin sections and improved fixation enabled us to observe it clearly in several micrographs.

It appears as a cluster of vesicles, arranged without order, rather large in size (60–300 Å) and of extremely variable but nonflat shape; *this cluster is always applied against the distal face* (by convention) of the stack of saccules. The chromophobe vesicles tend to separate from one another in the distal region of the cluster, and some micrographs give the impression that they gradually disperse into the surrounding cytoplasm. These vesicles, always only slightly osmiophilic, become increasingly pale as they move away from the stack of saccules. When contiguous, they may fuse (Plates I, III, and IV).

One can readily follow the transformation of the terminal saccules into chromophobe vesicles. They swell considerably while losing their osmiophilia, remain as such, or fragment into two, three, or four vesicles, generally of unequal size.

As a result of our investigations, the dictyosome appears as an organelle that powerfully produces various types of vesicles that differ in size and likely also in chemical nature. We recognize:

1. osmiophilic vesicles produced by “pearling” of the periphery of the saccules. All saccules, regardless of their position, produce osmiophilic vesicles;
2. large so‐called chromophobe vesicles deriving from the overall transformation of the distal saccules and constituting the chromophobe substance of the dictyosome of classical authors; and
3. some saccules located toward the distal end of the stack are subdivided into elements of approximately the same size and structure than osmiophilic vesicles.

It is not always easy to distinguish the third mode of formation from the second. However, true chromophobe vesicles are recognizable by their large size and their paleness. The distinction between these two modes of vesiculation is particularly clear in the idiozomes of certain aged spermatids of *Helix*.

Here is our interpretation of the Golgi apparatus: this cellular component consists of a variable number of dictyosomes (from one to several hundred). Each dictyosome exhibits a rigorously polarized structure (particularly evident in Zooflagellates and in the idiozome): one face of the stack, which we conventionally designate as distal, is in a state of perpetual destruction through chromophobe and total vesiculation of the saccules.

The opposite face, which we conventionally designate as proximal, consists of intact saccules with sharp contours. However, the most proximal saccules, in some sections, appear somewhat blurred and discontinuous. We have the impression, *though not the certainty*, that the proximal end of the stack is the site of continuous formation of new saccules, compensating for the destruction of distal elements that become the chromophobe substance.

All dictyosomes of the Zooflagellates examined by us present the same structure, the same modes of secretion, and their proximal extremity is in contiguous relation with the parabasal filament, an emanation of the centrosome.

For the first time, the undeniable morphological polarization of a cytoplasmic organelle and the close relationship between this polarization and the intrinsic activity of the said organelle are demonstrated.

The dictyosome is said to have the capacity to cleave and thus give rise to two or three daughter dictyosomes (dictyokinesis of Perroncito 1910). However, we have shown that, in several species of Zooflagellates, a dictyosome–parabasal complex forms de novo during binary cell division: one daughter individual retains the old (remodeled) dictyosome, while the other receives the new one (Grassé and Faure 1935, 1939)(*) Session of October 7, 1957. Ultrastructure, polarity, and reproduction of the Golgi apparatus. Grasse PP, *C R Hebd Seances Acad Sci*. 1957 Oct 14, 245 (16): 1278–1281.

We believe that the supposed cleavage and neoformation, rather than being contradictory, are in constant association. The cleavage is not as revealed by light microscopy. In reality, at a given moment, the proximal saccules in formation are discontinuous and, instead of a single stack, two, three, or more are constituted; the destruction of distal saccules by chromophobe vesiculation progressively leads to the disappearance of continuous saccules and, ultimately, the initial dictyosome is replaced by two or more dictyosomes which are not truly derived from it, but result from a neoformation within the cytoplasm itself, doubtless by local recruitment and transformation of certain macromolecules. Plate II clearly illustrates our interpretation.

**Explanation of the Plates**


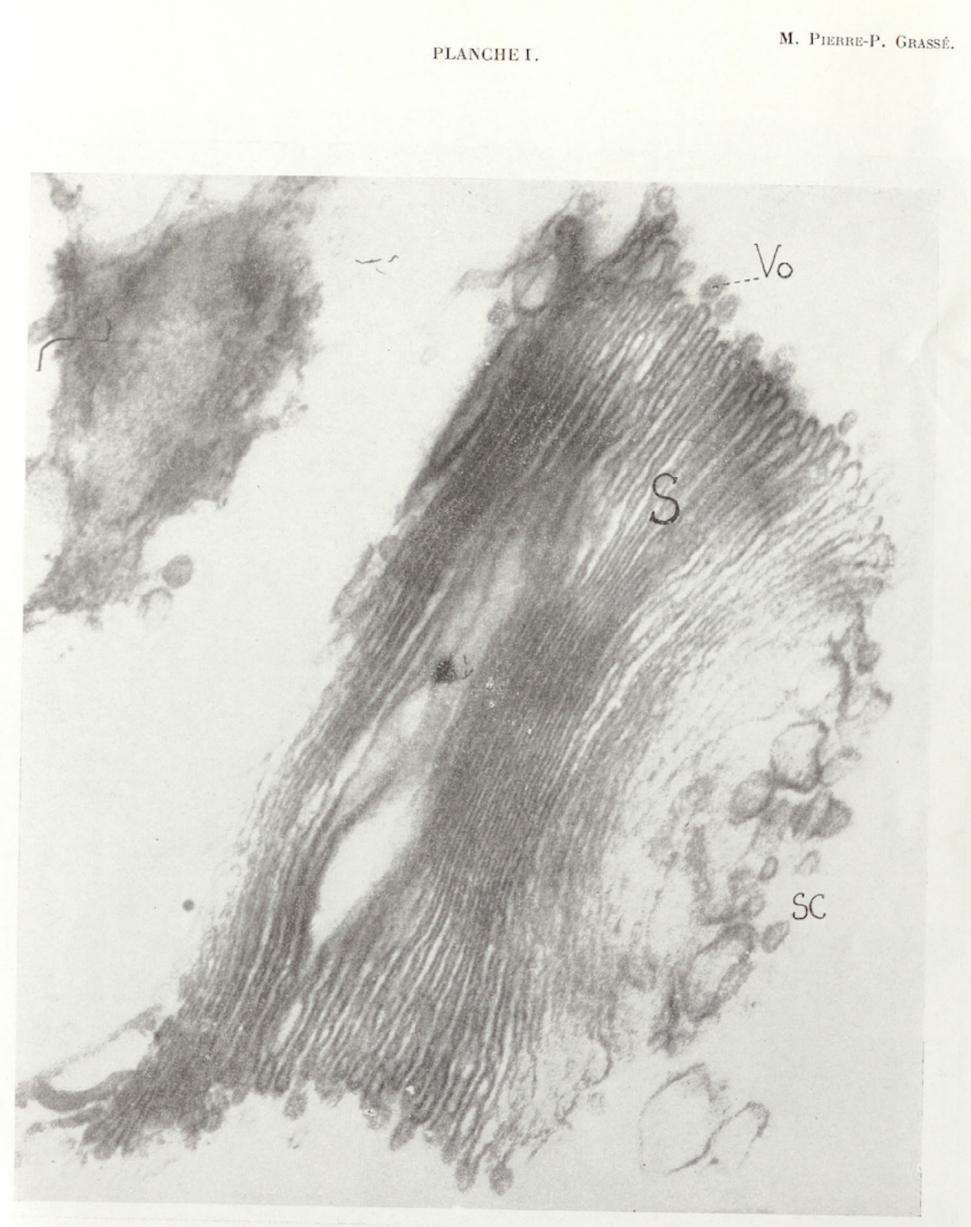


Golgi apparatus of a *Foaina* (Zooflagellate symbiont of *Calotermes flavicollis*) showing the stack of flat, closed saccules (*S*) that constitute it, the marginally positioned osmiophilic vesicles (*Vo*), and the large pale vesicles forming the chromophobe substance (*Sc*) at the distal extremity.

Direct photograph ×25,700; magnification ×64,200.


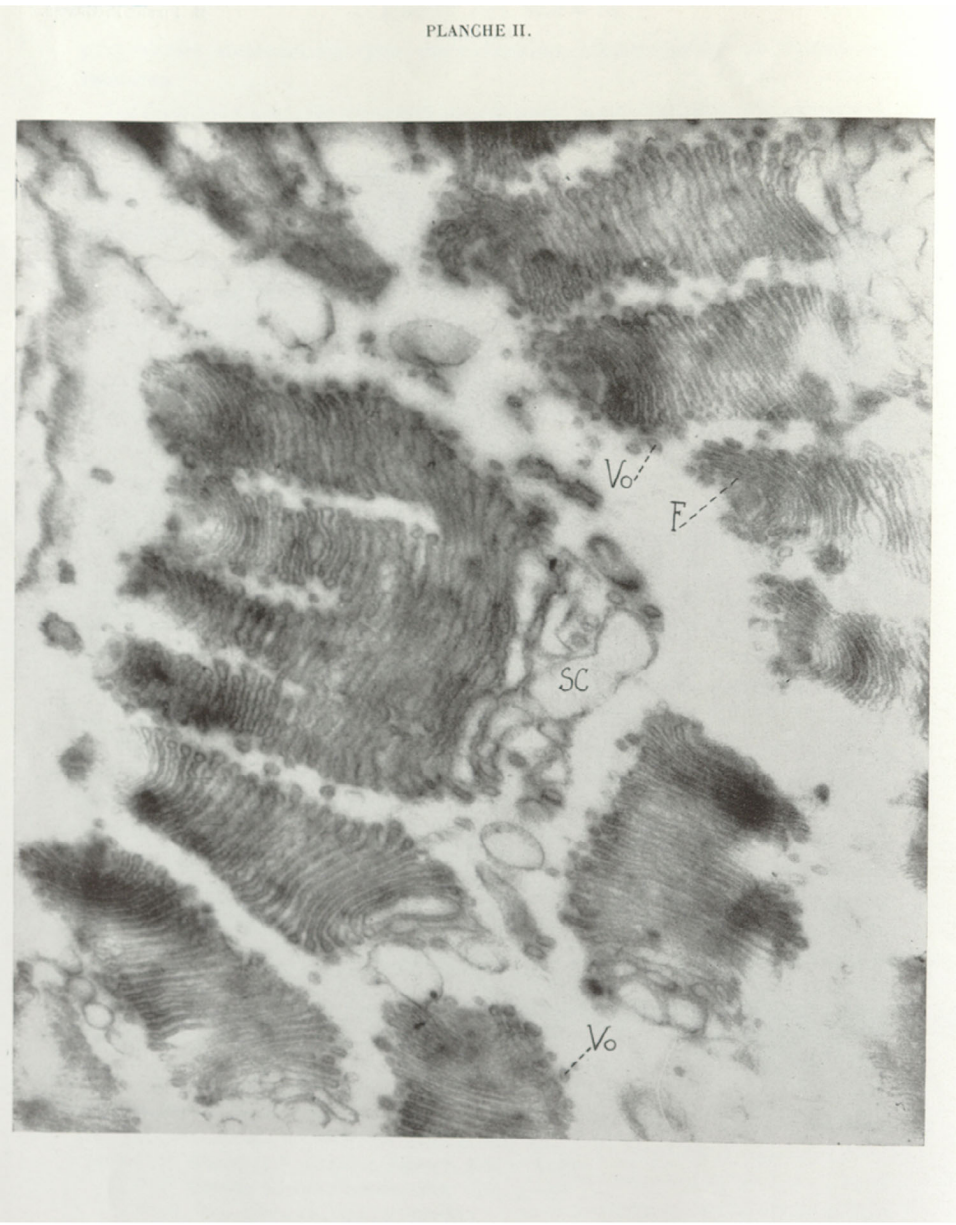


Section through the collar of dictyosomes of *Joenia annectens*, a Zooflagellate symbiont of *Calotermes flavicollis*. At the proximal extremity of several dictyosomes (= parabasal region) lies the parabasal filament (*F*); osmiophilic vesicles (*Vo*) surrounding the dictyosomes and the chromophobe substance (*Sc*) in large pale vesicles are clearly visible. One dictyosome, undergoing division, gives rise by substitution to four daughter elements. By mistake, the dashed line starting from the letter F has been extended one dash too far to the right.

Direct photograph ×19,000; magnification ×47,500.


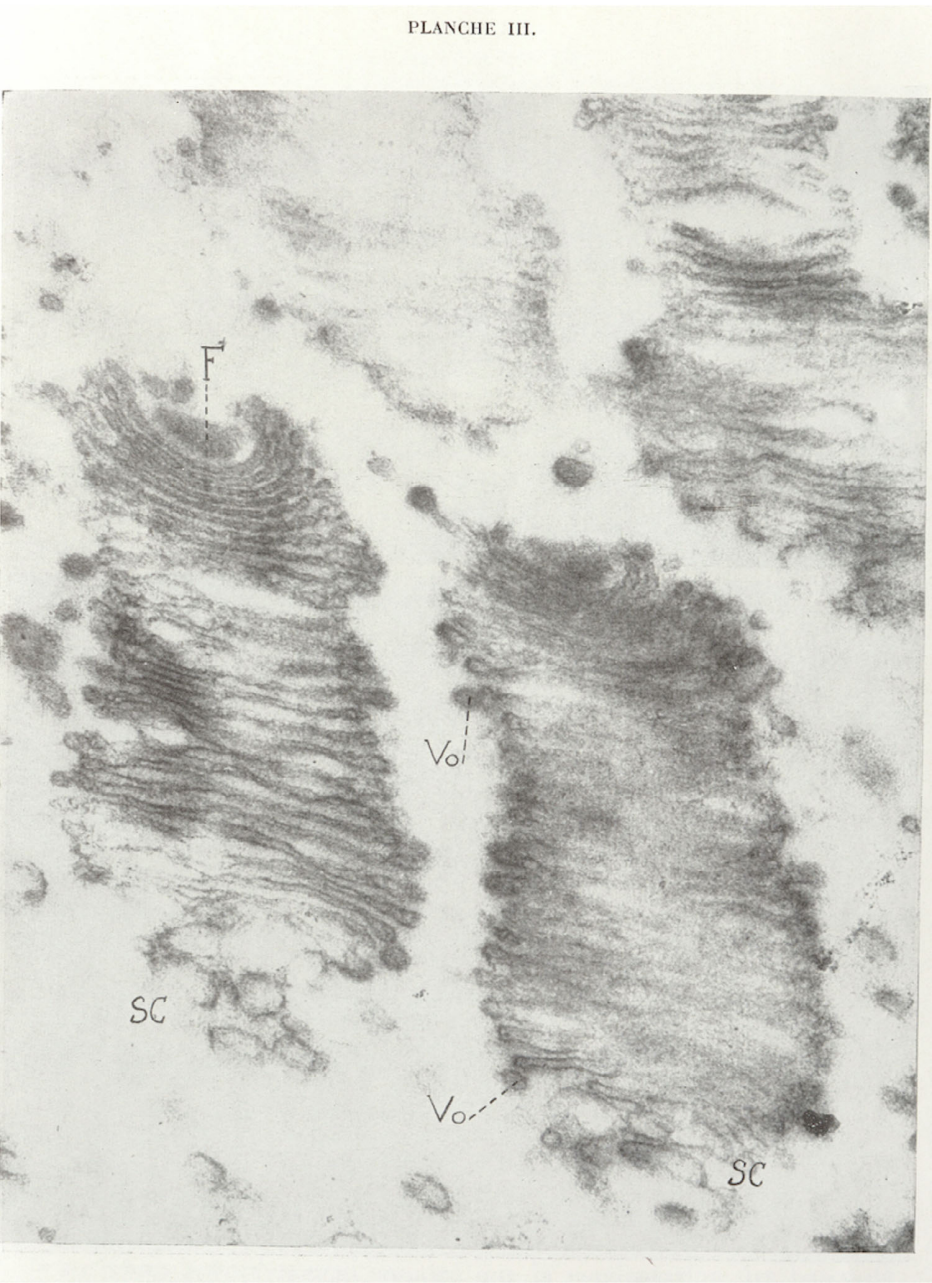


More highly magnified dictyosomes of *Joenia annectens*. *F*, parabasal filament; *Sc*, chromophobe substance; *Vo*, osmiophilic vesicles.

Direct photograph ×25,700; magnification ×64,250.

Dictyosome of *Foaina dogieli* with its centrosomal parabasal filament. The amount of chromophobe substance is considerable here. *Ch*, chromosome; *F*, parabasal filament; *M*, poorly fixed mitochondrion; *N*, nucleus; *Sc*, chromophobe substance; *Vo*, osmiophilic vesicles.

Direct photograph ×18,800; magnification ×47,500.


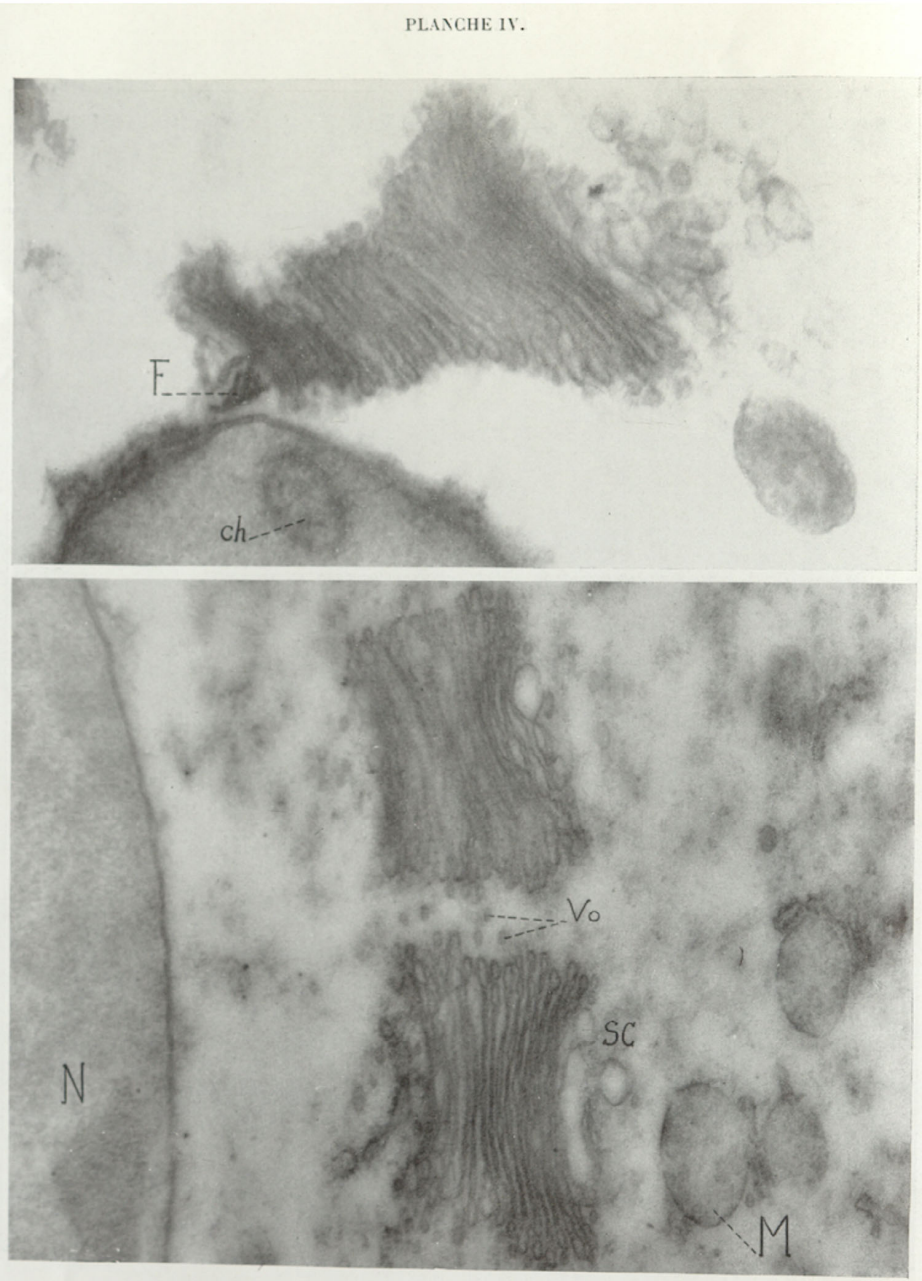


Transverse section of two long dictyosomes of *Trichonympha agilis*, a Zooflagellate symbiont of *Reticulitermes lucifugus*. *M*, mitochondria with irregular internal cristae; *N*, nucleus; *Sc*, scant chromophobe substance; *Vo*, numerous osmiophilic vesicles.

Direct photograph ×18,800; magnification ×47,000.

## Footnotes

(*) P.‐P. Grassé, Ultrastructure, polarity, and reproduction of the Golgi apparatus, Session of the French Academy of Sciences of October 7, 1957.
